# The Elastin-Derived Peptide VGVAPG Does Not Activate the Inflammatory Process in Mouse Cortical Astrocytes In Vitro

**DOI:** 10.1007/s12640-019-00114-x

**Published:** 2019-11-05

**Authors:** Konrad A. Szychowski, Jan Gmiński

**Affiliations:** 1 Department of Clinical Biochemistry and Laboratory Diagnostics, Institute of Medicine, University of Opole, Oleska 48, 45-052 Opole, Poland; 2 Department of Lifestyle Disorders and Regenerative Medicine, University of Information Technology and Management in Rzeszow, Sucharskiego 2, 35-225 Rzeszow, Poland

**Keywords:** Elastin-derived peptides, VGVAPG, Astrocyte, Inflammation

## Abstract

During vascular aging or in pathological conditions in humans, elastin is degraded and its by-products, the elastin-derived peptides (EDPs), enter the blood circulation. EDPs may be detected in the serum of healthy subjects or people who suffered a stroke. Moreover, recent evidence suggests a potential role of inflammatory mechanisms in neurological conditions, which are usually not categorized as inflammatory. Therefore, the present in vitro study was conducted to investigate the impact of the VGVAPG peptide on the activation of inflammatory process in mouse primary astrocytes, which were maintained in phenol red-free DMEM/F12 supplemented with 10% fetal bovine serum. The cells were exposed to VGVAPG or VVGPGA peptides for 24 and 48 h; this was followed by the determination of the activity of caspase-1 and levels of SOD, CAT, PPARγ, NF-κB, IL-1β, and IL-1βR1. Furthermore, rosiglitazone—a PPARγ agonist—was applied. Our study pioneered the finding that the VGVAPG peptide increases caspase-1 activity in astrocytes in vitro. The VGVAPG peptide simultaneously decreases the release of IL-1β into the cell-culture medium from astrocytes*.* The ELISA method revealed that the VGVAPG peptide increases the protein expression of SOD1 whereas it decreases the expression of IL-1βR1, CAT, and NF-κB. Therefore, the available data suggest that the VGVAPG peptide (concentration 10 nM) synergistically acts with agonists of PPARγ in mouse astrocytes. However, given the lack of sufficient data to explain the molecular mechanism of action of the VGVAPG peptide in the nervous system, more studies in this area are necessary.

## Introduction

Elastin is mainly responsible for tissue elasticity and is an insoluble component of elastic fibers in the skin, lung, and arteries (Mecham 2012). Elastin can be degraded by several enzymes known as elastases. In humans, elastin is degraded during vascular aging or in pathological conditions, following which the by-products—elastin-derived peptides (EDPs)—enter and persist in the blood circulation (Gayral et al. 2014). In research published so far, high elastase expression and activity have been noted in different immune cells such as macrophages, leukemic neutrophils, or microglia (Gudgeon et al. 2013; Liu et al. 2013). Moreover, in the aging brain, matrix metalloproteinases (MMPs) from microglia can degrade elastin, facilitate the migration of inflammatory cells in tissues, and thereby modulate their inflammatory activity (Liu et al. 2013). EDPs can be detected in the serum of healthy subjects, and their levels increase in patients after a stroke (Nicoloff et al. 2008; Tzvetanov et al. 2008). Moreover, elevated levels of EDPs are detected in type 2 diabetes mellitus (T2DM), systemic sclerosis, cancers, and pulmonary diseases (Nicoloff et al. 2010; Hong et al. 2012; Skjøt-Arkil et al. 2013; Nikolov et al. 2014). Furthermore, several papers have suggested that the presence of EDPs is a trigger mechanism, which can stimulate inflammatory processes in the human abdominal aorta, *ligamentum flavum* cells, and synovial cells (Satta et al. 1998; Chao et al. 2012; Kobayashi et al. 2017). In accordance with the principle of positive feedback, EDPs can further generate more EDPs, and the whole process is accelerating (Dale et al. 2016; Kobayashi et al. 2017). This positive feedback loop, also known as the *Snowball phenomenon*, leads to chronic inflammation that is associated with the abovementioned diseases (cancer, atherosclerosis, T2DM) (Coussens and Werb 2012; Calle and Fernandez 2012; Libby 2012). However, there is much evidence to suggest a potential role of inflammatory mechanisms even in neurological conditions, such as Alzheimer’s disease, Parkinson’s disease, Huntington’s disease, amyotrophic lateral sclerosis, stroke, and traumatic brain injuries (Degan et al. 2018).

Different authors have opined that interleukin-1 beta (IL-1β) is a key pro-inflammatory cytokine in the central nervous system (CNS) (Moynagh 2005). IL-1β stimulates the production of adhesion molecules and chemokines in astrocytes (Moynagh et al. 1994; Bourke and Moynagh 1999). IL-1β is formed when its inactive precursor pro-IL-1β is activated by limited proteolysis through the interleukin-1-beta–converting enzyme (ICE), which is currently known as caspase-1 (Scheer 2013). At present, it is generally accepted that caspase-1 is not involved in apoptosis, but that its role is limited to the inflammatory process in immune cells (Miao et al. 2011; Strowig et al. 2012; Sollberger et al. 2014). However, caspase-1 can be activated in many nonimmune cell types, which include keratinocytes, astrocytes, hepatocytes, and cardiomyocytes, wherein IL-1β and − 18 are not produced at significant levels and, therefore, suggests alternative functions for caspase-1 (Yazdi et al. 2010; Ganz et al. 2011). Several studies have shown caspase-1 to be a regulator of cellular responses to stress through the regulation of cytoprotective responses, tissue repair, and autophagy (Keller et al. 2008; Groß et al. 2012; Sun et al. 2013; Saitoh and Akira 2016). Nevertheless, caspase-1 was claimed to be a requirement for apoptosis in neuronal or endothelial cells by some authors (Friedlander 2003; Sollberger et al. 2014). This particular aspect of caspase-1 function may be especially important in hepatocytes, neurons, astrocytes, and cardiomyocytes, where cell and tissue stresses, aging, or damage can be the source of EDPs being released from the extracellular matrix (Ganz et al. 2011; Kaczmarek et al. 2013; Sun and Scott 2016).

The transcription nuclear factor kappa-light-chain-enhancer of activated B cells (NF-κB) is a crucial mediator in the IL-1 signaling pathway and acts as a major inductor in the production of adhesion molecules and cytokines (Moynagh 2005). Moreover, the presence of IL-1β stimulates the activation of the NF-κB pathway, which then initiates the production of increased amounts of IL-1β (Liu et al. 2017). However, rosiglitazone—a peroxisome proliferator-activated receptor γ (PPARγ) agonist (and other synthetic, e.g., thiazolidinediones, or endogenous, e.g., 15-Deoxy-Delta12,14-prostaglandin J2 [15d-PGJ2], PPARγ agonists)—can interrupt the positive feedback loop of IL-1β through the increased production of the interleukin-1 receptor antagonist (IL-1Ra) in rat synovial fibroblasts (Moulin et al. 2005). Moreover, PPARγ agonists and glucocorticoids inhibit the DNA-binding activity of NF-κB and thereby disrupt the NF-κB pathway (Remels et al. 2009; Liu et al. 2017). However, recent data shows that PPARγ is essential for the proper activation of the inflammatory process and regulation of expression antioxidant enzymes such as superoxide dismutase 1 (SOD1) and catalase (CAT) (Kim and Yang 2013; Weber et al. 2018).

The present in vitro study was conducted to investigate the impact of the VGVAPG peptide and rosiglitazone on the activation of inflammatory process in mouse primary astrocytes.

## Materials and Methods

### Reagents

Dulbecco’s Modified Eagle’s Medium/Hams F-12 (DMEM/F12) without phenol red as well as trypsin, rosiglitazone, penicillin, streptomycin, amphotericin B, 3-[(3-cholamidopropyl)dimethylammonio]-1-propanesulfonate (CHAPS), 4-(2-hydroxyethyl)piperazine-1-ethanesulfonic acid (HEPES), ethylenediaminetetraacetic acid (EDTA), DL-dithiothreitol (DTT), N-α-acetyl-Tyr-Val-Ala-Asp-p-nitroanilide (Ac-YVAD-pNA), and dimethyl sulfoxide (DMSO) were purchased from Sigma-Aldrich (St. Louis, MO, USA). The VGVAPG and VVGPGA peptides were synthesized by LipoPharm.pl (Gdańsk, Poland). Charcoal/dextran-treated FBS was purchased from EURx (Gdańsk, Poland). ELISA kits for PPARγ (M0893), SOD1 (M2398), IL-1β (M0037), IL-1R1 (M0017), and CAT (M2605) were purchased from Elabscience Biotechnology (Wuhan, China). NF-κB (EM1230) was purchased from Wuhan Fine Co., Ltd. (Wuhan, China). Stock solutions of the VGVAPG and VVGPGA peptides were prepared in DMSO and, then, added to the DMEM/F12 medium. The final concentration of DMSO in the culture medium was always 0.1%.

### Astrocyte-Enriched Cell Culture

The experiments were conducted on mouse astrocyte cell cultures. All experimental procedures were approved by a Bioethics Commission (approval no. 46/2014; First Local Ethical Committee on Animal Testing at the Jagiellonian University in Krakow), as compliant with the laws of the European Union. The cell cultures were prepared from the fetuses of pregnant, female Swiss mice on Day 17/18 of gestation. After the isolation and digestion processes, the cells were centrifuged and the pellet was suspended in DMEM/F12 1:1, without phenol red, that was supplemented with 10% FBS, 100 U/mL penicillin, 0.10 mg/mL streptomycin, and 250 ng/mL amphotericin B. Isolation was undertaken according to a previously described method that allows the separation of an almost pure culture of astrocytes (>98% astrocytes) (Szychowski et al., 2019b, supplementary data). The cultures of glial cells were maintained at 37 °C in a humidified atmosphere containing 5% CO_2_. In the logarithmic phase after reaching 90% confluence, the cells were trypsinized with 0.25% trypsin/0.05% EDTA and passaged onto an experimental plate. The astrocytes were respectively seeded at densities of 5 × 10^5^ per well in a 96-well plate for colorimetric analysis and 60 × 10^5^ per well in a six-well plate for protein analysis. The culture medium was changed prior to the experiment with the VGVAPG and VVGPGA peptides and tool compound selected for this study.

### Caspase-1 Activity

Caspase-1 activity was assessed according to the method described by Nicholson et al. (1995), with some modifications. The cultured glial cells were lysed using lysis buffer (50 mM HEPES, pH 7.4, 100 mM NaCl, 0.1% CHAPS, 1 mM EDTA, 10% glycerol, and 10 mM DTT) at 10 °C for 10 min. After initial incubation, the lysates were incubated with the caspase-1 substrate Ac-YVAD-pNA at 37 °C. After 30 min, absorbance of the lysates at 405 nm was measured on a microplate reader (FilterMax F5 Multi-Mode microplate reader). The quantity of colorimetric product was continuously monitored for 120 min.

### ELISA for SOD, CAT, PPARγ, NF-κB, IL-1β, and Interleukin 1 Beta Receptor 1 (IL-1βR1)

The levels of SOD1 (M2398), CAT (M2605), PPARγ (M0893), NF-κB (EM1230), IL-1β (M0037), and IL-1R1 (M0017) (purchased from Elabscience Biotechnology or Wuhan Fine Co., Ltd., Wuhan, China) proteins were determined through ELISA after 24 or 48 h of treatment with 10 nM VGVAPG, 10 nM VVGPGA or co-treatment with 10 μM rosiglitazone. The expression of SOD, CAT, PPARγ, NF-κB, and IL-1βR1 were determined in cell-culture lysate, whereas IL-1β release was measured in the cell-culture medium. These proteins were specifically detected with ELISA and subsequently subjected to quantitative sandwich enzyme immunoassay, which was conducted according to the manufacturer’s instructions (Elabscience Biotechnology, Wuhan, China). Briefly, a 96-well plate was pre-coated with monoclonal antibodies specific to SOD, CAT, PPARγ, NF-κB, IL-1β, and IL-1βR1. Standards and the collected cell extracts were added to the wells and incubated for 90 min at 37 °C. Next, the liquid was removed, and 100 μL biotinylated detection antibodies were added to the cultures for 60 min. We washed the cells three times to remove any unbound substances and then added horseradish peroxidase-conjugated avidin. The cells were washed again, and 90 μL substrate solution was added to the wells for 15 min. Then, 50 μL stop solution was added to terminate the reaction and absorbance was measured at 450 nm; the value obtained was proportional to the amount of SOD, CAT, PPARγ, NF-κB, IL-1β, and IL-1βR1, respectively. The total protein concentration was determined in triplicate in each sample by using a Thermo Fisher NanoDrop device.

### Statistical Analysis

Data are presented as means ± SD of three independent experiments. Each treatment was repeated six times (*n* = 6) and measured in triplicate. Experimental data were analyzed with one-way analysis of variance (ANOVA) followed by Tukey’s multiple comparison test. Statistical significance was determined at ***p < 0.001, **p < 0.01, and *p < 0.05 vs the control.

## Results

### Caspase-1 Activity

**Fig. 1:**
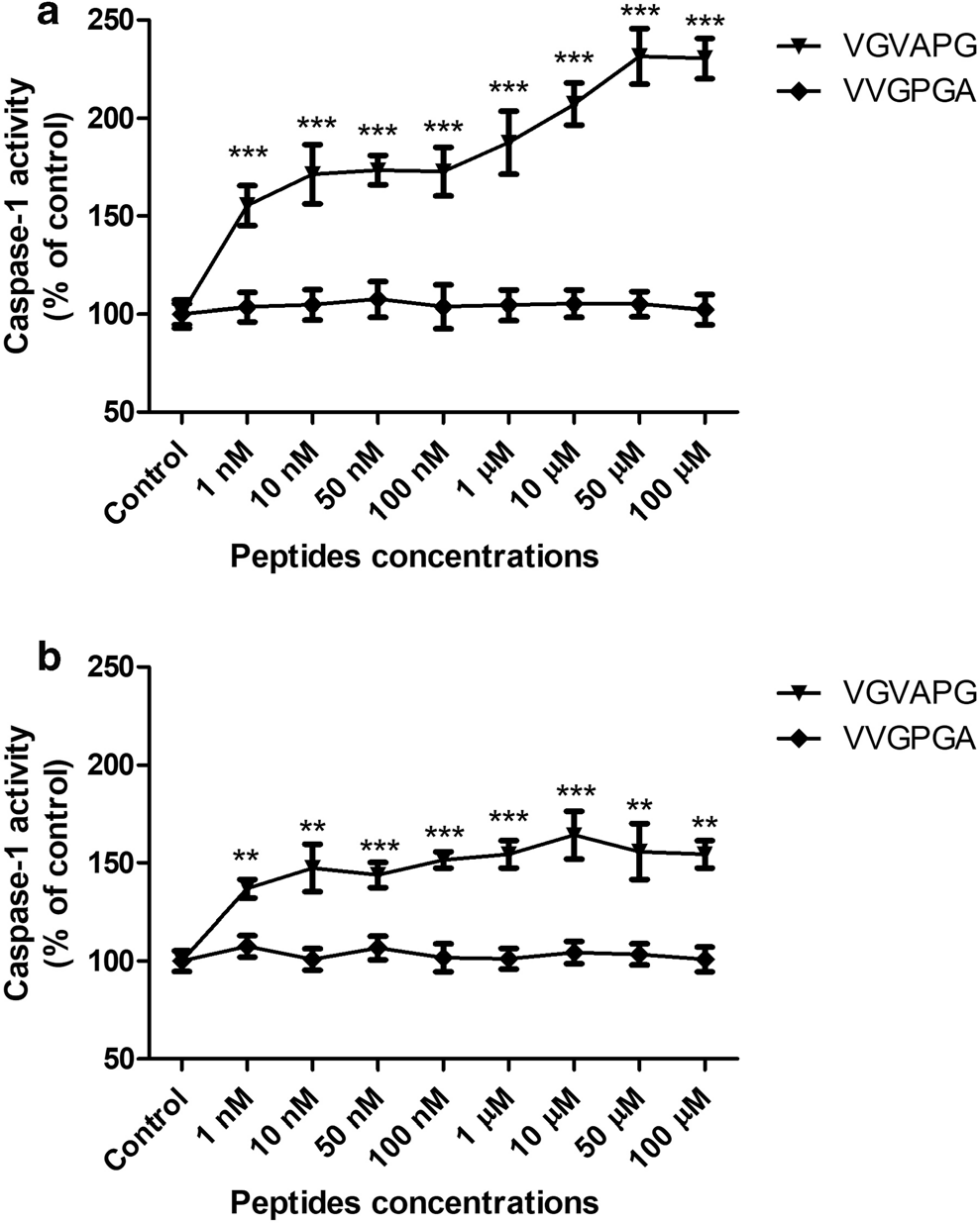
Effect of the VGVAPG and VVGPGA peptides on the activity of caspase-1 after 24 (**a**) and 48 (**b**) h in mouse primary astrocytes in vitro. Data are expressed as means ± SD of three independent experiments, each of which comprised six replicates per treatment group. **p < 0.01 and ***p < 0.001, vs the control cells

After 24-h exposure of mouse astrocytes to increasing concentrations (1–100 nM and 1–100 μM) of the VGVAPG peptide, the activity of caspase-1 increased at all of the studied concentrations (increase by 55.35–130.21% vs control). The VVGPGA peptide does not affect caspase-1 activity within cells (Fig. 1a). Similarly, after 48-h exposure of mouse astrocytes to increasing concentrations (1–100 nM and 1–100 μM) of the VGVAPG peptide, the activity of caspase-1 increased at all of the studied concentrations (increase by 36.83–26.31% vs control). The VVGPGA peptide does not affect caspase-1 activity in cells (Fig. 1b).

**Fig. 2:**
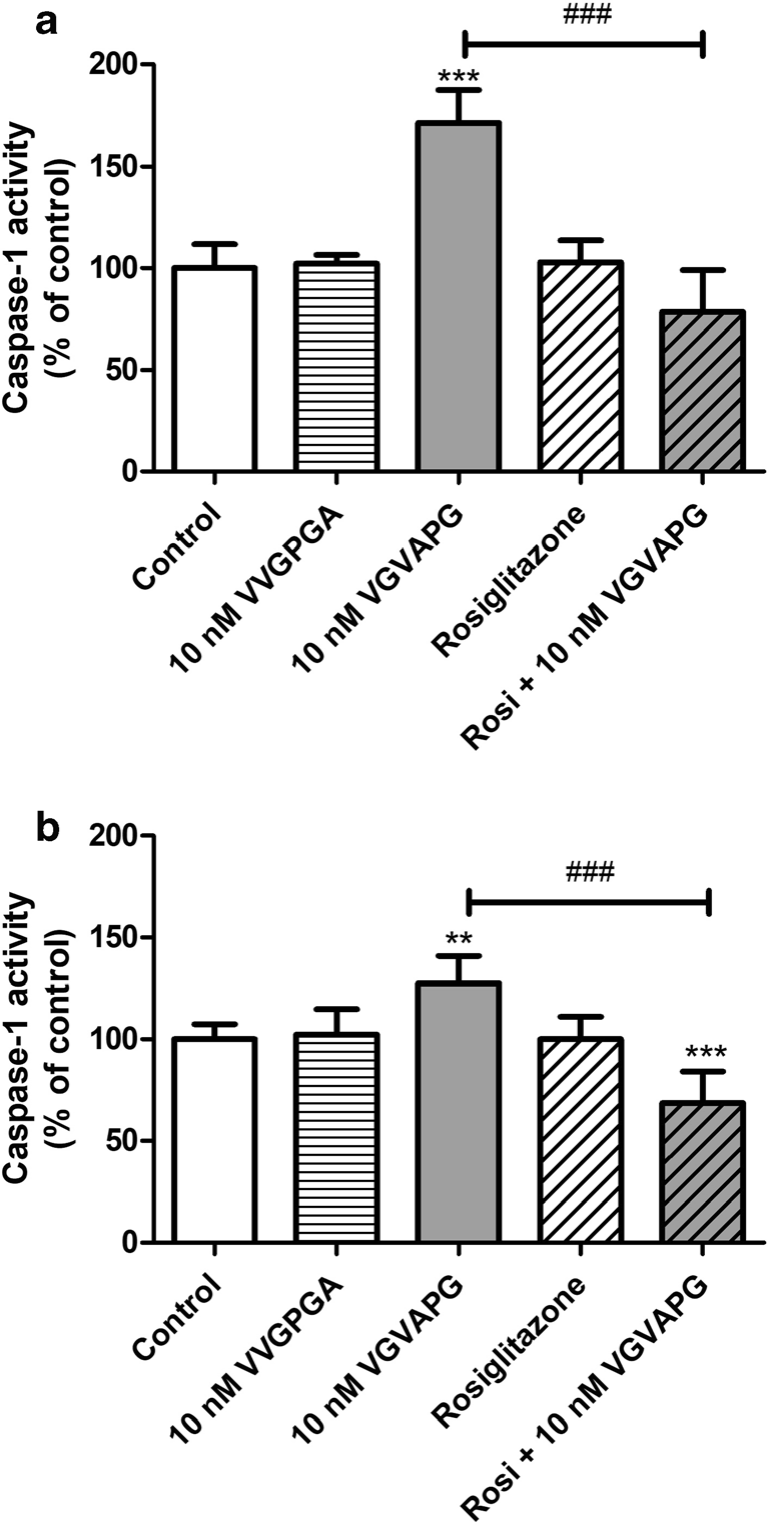
Effect of the 10 nM VGVAPG and VVGPGA peptides and 10 μM rosiglitazone on the activity of caspase-1 after 24 (**a**) and 48 (**b**) h in mouse primary astrocytes in vitro. Data are expressed as means ± SD of three independent experiments, each of which comprised six replicates per treatment group. **p < 0.01 and ***p < 0.001, vs the control cells. ^###^*p* < 0.001 vs the group treated with the VGVAPG peptide

Following 24-h exposure of mouse astrocytes to 10 nM VGVAPG peptide, the activity of caspase-1 increased by 71.41%, compared to the control. Treatment of astrocytes by 10 μM rosiglitazone did not affect the activity of caspase-1. Moreover, in cells co-treated with 10 nM VGVAPG and 10 μM rosiglitazone, we did not observe changes in the activity of caspase-1 (Fig. 2a). After 48-h exposure of mouse astrocytes to 10 nM VGVAPG peptide, the activity of caspase-1 increased by 27.45%, compared to the control. Similarly, treatment of these astrocytes with 10 μM rosiglitazone after 48-h exposure did not affect the activity of caspase-1. However, cell co-treatment with 10 nM VGVAPG and 10 μM rosiglitazone decreased the activity of caspase-1 by 31.38%, compared to the control (Fig. 2b).

### Protein Expression ELISA Assay

#### Expression of IL-1β and IL-1βR1

**Fig. 3:**
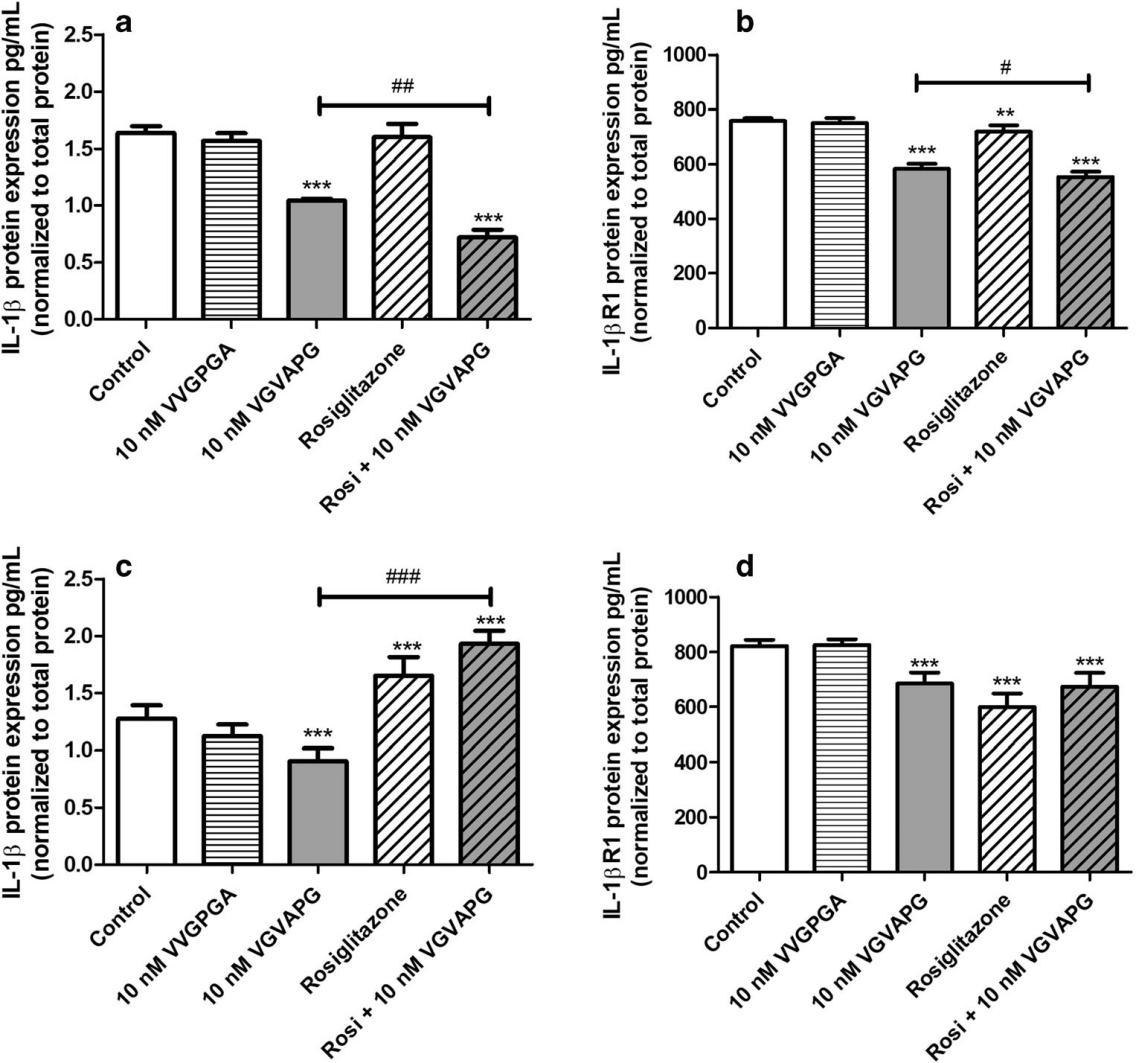
Effect of the 10 nM VGVAPG and VVGPGA peptides and 10 μM rosiglitazone on the protein expression of IL-1β and IL-1βR1 after 24 (**a**, **b**) and 48 (**c**, **d**) h in mouse primary astrocytes in vitro. Data are expressed as means ± SD of three independent experiments, each of which comprised six replicates per treatment group. **p < 0.01 and ***p < 0.001, vs the control cells. ^##^*p* < 0.01 and ^###^*p* < 0.001, vs the group treated with the VGVAPG peptide

After 24-h exposure of mouse astrocytes to 10 nM VGVAPG peptide, we observed a decrease in the release of IL-1β into the cell-culture medium (decrease by 0.60 pg/mL vs control). Rosiglitazone does not affect the release of IL-1β into the cell-culture medium. In cells co-treated with 10 nM VGVAPG and 10 μM rosiglitazone, the release of IL-1β into the culture medium decreased by 0.33 pg/mL, compared to the group treated with 10 nM VGVAPG (Fig. 3a). Following 48-h exposure of mouse astrocytes to 10 nM VGVAPG peptide, the release of IL-1β into the cell-culture medium decreased by 0.37 pg/mL, compared to the control. In astrocytes treated with 10 μM rosiglitazone, we observed an increase in the release of IL-1β by 0.38 pg/mL, compared to the control. In cells co-treated with 10 nM VGVAPG and 10 μM rosiglitazone, the release of IL-1β increased by 1.03 pg/mL as compared to the group treated with 10 nM VGVAPG (Fig. 3c).

Furthermore, following 24-h exposure of mouse astrocytes to 10 nM VGVAPG peptide, we observed a decrease in the expression of IL-1βR1 (decrease by 175.05 pg/mL vs control). Similarly, in astrocytes treated with 10 μM rosiglitazone, we observed a decrease in the expression of IL-1βR1 (by 40.23 pg/mL vs control). In cells co-treated with 10 nM VGVAPG and 10 μM rosiglitazone, the expression of IL-1βR1 decreased by 30.66 pg/mL compared to the group treated with 10 nM VGVAPG (Fig. 3b). Moreover, after 48-h exposure of mouse astrocytes to 10 nM VGVAPG peptide, the expression of IL-1βR1 decreased by 136.37 pg/mL, compared to the control. In astrocytes treated with 10 μM rosiglitazone, we observed a decrease in IL-1βR1 expression of 221.69 pg/mL, compared to the control. After 48-h in cells co-treated with 10 nM VGVAPG and 10 μM rosiglitazone, the expression of IL-1βR1 did not change when compared to the group treated with 10 nM VGVAPG (Fig. 3d).

#### Expression of SOD and CAT

**Fig. 4:**
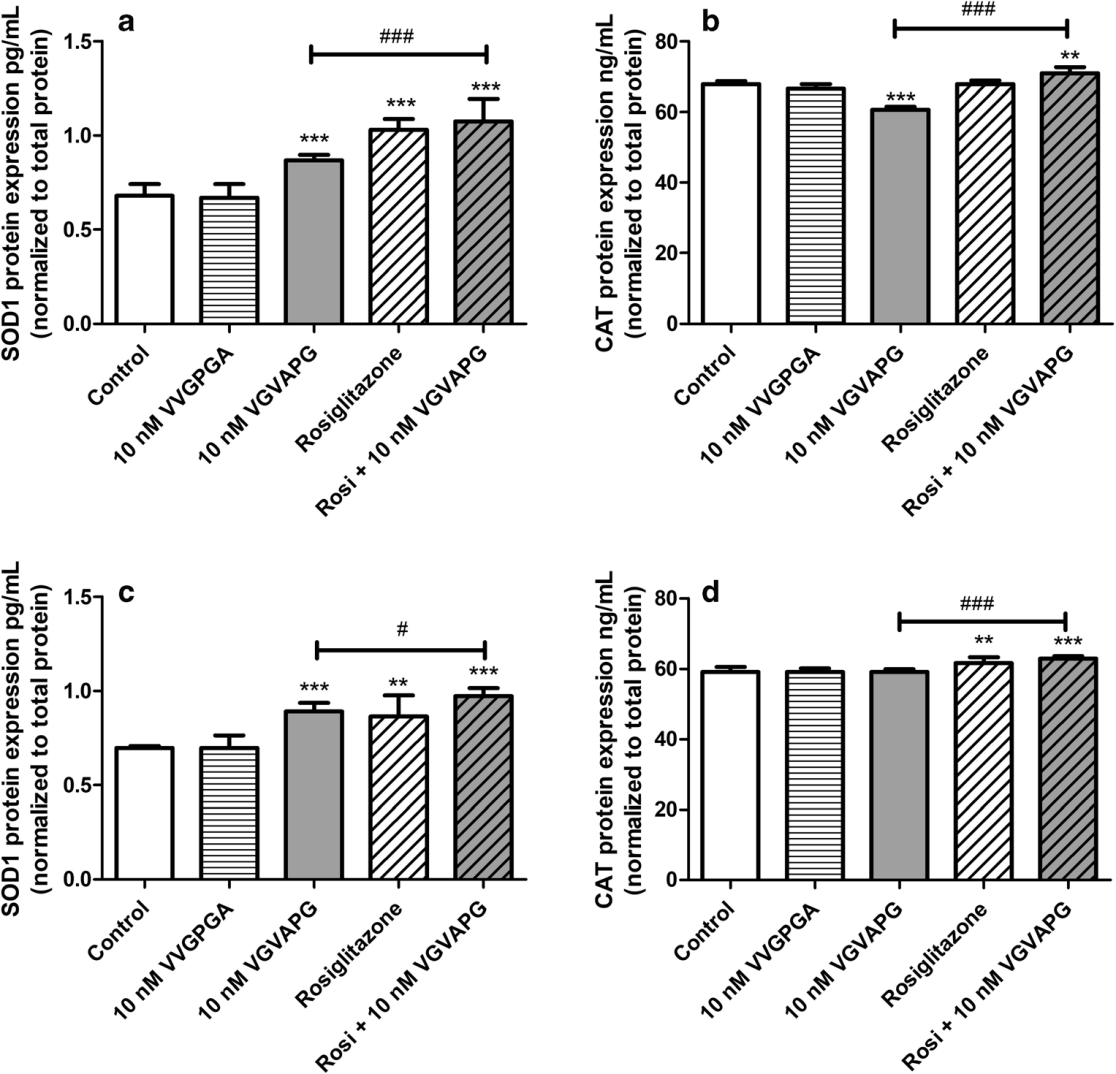
Effect of the 10 nM VGVAPG and VVGPGA peptides and 10 μM rosiglitazone on protein expression of SOD1 and CAT after 24 (**a**, **b**) and 48 (**c**, **d**) h in mouse primary astrocytes in vitro. Data are expressed as means ± SD of three independent experiments, each of which comprised six replicates per treatment group. **p < 0.01 and ***p < 0.001, vs the control cells. ^#^*p* < 0.05 and ^###^*p* < 0.001, vs the group treated with the VGVAPG peptide

After 24-h exposure of mouse astrocytes to 10 nM VGVAPG peptide, the expression of SOD1 increased by 0.19 pg/mL, compared to the control. Moreover, treatment of astrocytes with 10 μM rosiglitazone increased the expression of SOD1 by 0.36 pg/mL, compared to the control. In cells co-treated with 10 nM VGVAPG and 10 μM rosiglitazone, the expression of SOD1 increased by 0.21 pg/mL when compared to the group treated with 10 nM VGVAPG (Fig. 4a). After 48-h exposure of mouse astrocytes to 10 nM VGVAPG peptide, the expression of SOD1 increased by 0.20 pg/mL, compared to the control. Treatment of astrocytes with 10 μM rosiglitazone increased the expression of SOD1 by 0.17 pg/mL, compared to the control. In cells co-treated with 10 nM VGVAPG and 10 μM rosiglitazone, the expression of SOD1 increased by 0.08 pg/mL when compared to the group treated with 10 nM VGVAPG (Fig. 4c).

After 24-h exposure of mouse astrocytes to 10 nM VGVAPG peptide, the expression of CAT decreased by 7.14 ng/mL, compared to the control. Treatment of astrocytes with 10 μM rosiglitazone did not change the expression of CAT. In cells co-treated with 10 nM VGVAPG and 10 μM rosiglitazone, the expression of CAT increased by 10.27 ng/mL as compared to that in the group treated with 10 nM VGVAPG (Fig. 4b). After 48-h treatment of astrocytes with 10 μM rosiglitazone, the expression of CAT increased by 2.55 ng/mL, compared to the control. The exposure of astrocytes to 10 nM VGVAPG did not change the expression of CAT. In the group co-treated with 10 nM VGVAPG and 10 μM rosiglitazone, the expression of CAT increased by 3.78 ng/mL as compared to the group treated with 10 nM VGVAPG (Fig. 4d).

#### Expression of PPARγ and NF-κB

**Fig. 5:**
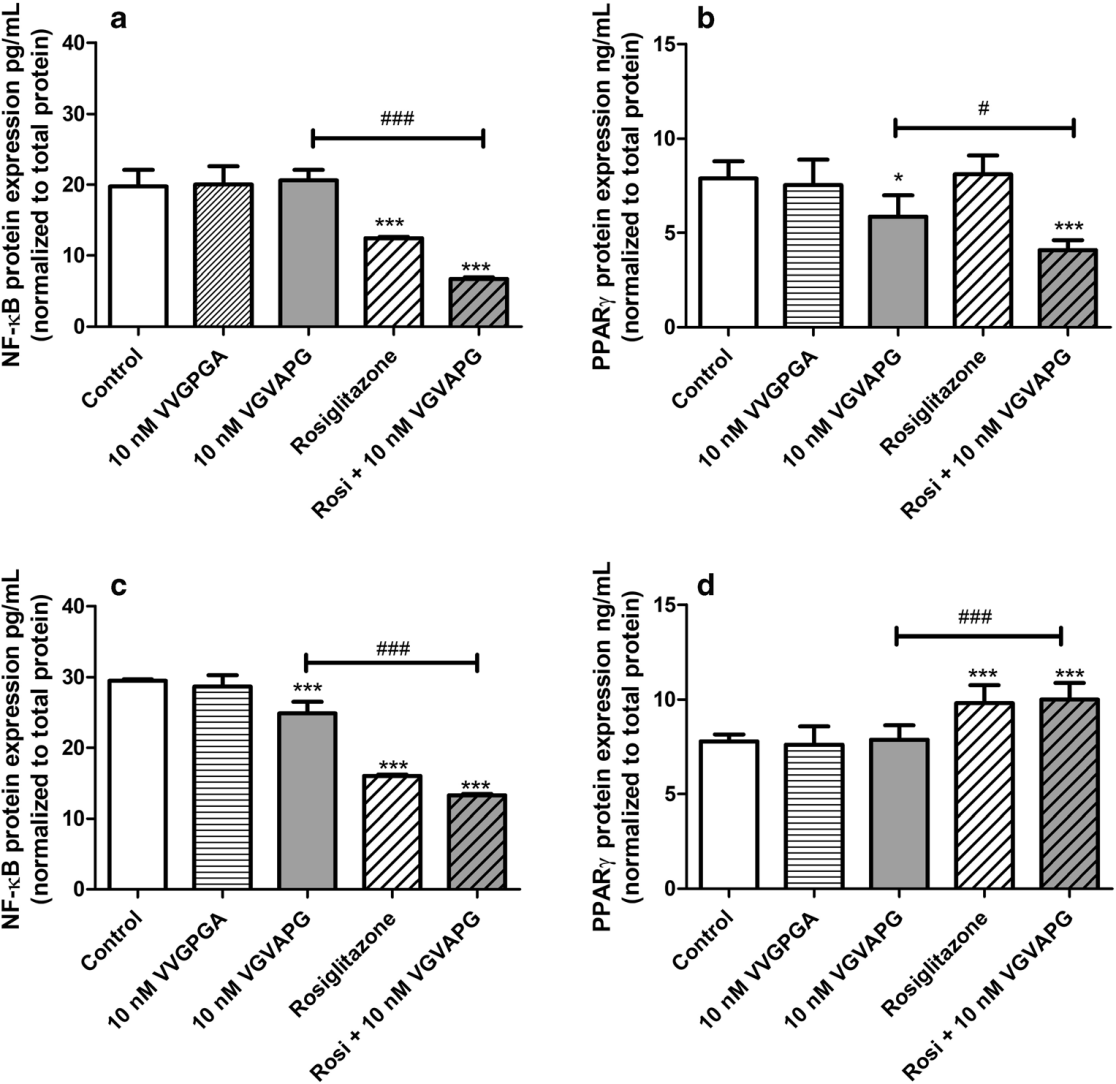
Effect of the 10 nM VGVAPG and VVGPGA peptides and 10 μM rosiglitazone on the protein expression of NF-κB and PPARγ after 24 (**a**, **b**) and 48 (**c**, **d**) h in mouse primary astrocytes in vitro. Data are expressed as means ± SD of three independent experiments, each of which comprised six replicates per treatment group. *p < 0.05 and ***p < 0.001, vs the control cells. ^#^*p* < 0.05 and ^###^*p* < 0.001, vs the group treated with the VGVAPG peptide

Following 24-h exposure of mouse astrocytes to 10 nM VGVAPG peptide, we did not observe any changes in the expression of NF-κB. Treatment of astrocytes with 10 μM rosiglitazone decreased the expression of NF-κB by 7.28 pg/mL, compared to the control. In cells co-treated with 10 nM VGVAPG and 10 μM rosiglitazone, the expression of NF-κB decreased by 13.89 pg/mL when compared to that in the group treated with 10 nM VGVAPG (Fig. 5a). After 48-h exposure of mouse astrocytes to 10 nM VGVAPG peptide, the expression of NF-κB decreased by 4.61 pg/mL, compared to the control. Furthermore, treatment of astrocytes by 10 μM rosiglitazone decreased the expression of NF-κB by 13.50 pg/mL, compared to the control. In cells co-treated with 10 nM VGVAPG and 10 μM rosiglitazone, the expression of NF-κB decreased by 11.63 pg/mL as compared to the group that was treated with 10 nM VGVAPG (Fig. 5c).

After 24-h exposure of mouse astrocytes to 10 nM VGVAPG peptide, the expression of PPARγ decreased by 2.03 ng/mL, compared to the control. Treatment of astrocytes with 10 μM rosiglitazone did not change the expression of PPARγ. In cells co-treated with 10 nM VGVAPG and 10 μM rosiglitazone, the expression of PPARγ decreased by 1.78 ng/mL as compared to the group that was treated with 10 nM VGVAPG peptide (Fig. 5b). After 48-h exposure of mouse astrocytes to 10 nM VGVAPG peptide, the expression of PPARγ did not change, compared to the control. Treatment of astrocytes by 10 μM rosiglitazone increased the PPARγ expression by 2.04 ng/mL, compared to the control. In cells co-treated with 10 nM VGVAPG peptide and 10 μM rosiglitazone, the expression of PPARγ increased by 2.12 ng/mL as compared to the group that was treated with 10 nM VGVAPG (Fig. 5d).

## Discussion

Increased activity of caspase-1 is believed to lead to the initiation of the cell/tissue inflammatory process (Scheer 2013). Our experiments in this study generated the novel finding that the VGVAPG peptide at all of the studied concentrations (1 nM to 100 μM) increases the activity of caspase-1 in mouse primary astrocytes in vitro. In the experiments we conducted, the VVGPGA peptide that was used as a control did not activate caspase-1 in the cells. To date, there are no data in the literature on the activity of caspase-1 after the stimulation of astrocytes or other cell types with EDPs. However, different authors have consistently maintained that EDPs induced the production and/or secretion of IL-1α, IL-1β, and IL-6 in *ligamentum flavum* cells, synovial cells, and melanoma cell lines; therefore, we can assume that caspase-1 should be activated by EDPs in these cells (Satta et al. 1998; Debret et al. 2006; Kobayashi et al. 2017). It is well documented that the mechanism of action of the VGVAPG peptide involves the hormesis phenomenon. The effect of the action of the VGVAPG peptide depends on the concentration that is used. The reaction curve takes the shape of a non-linear dose–response, usually with a “U”, “N”, or sinusoid shape (Senior et al. 1984; Fujimoto et al. 2000). Interestingly, in our experiments, we show that an increase in the activity of caspase-1 is correlated with the concentration of the VGVAPG peptide that is used. The data from our experiments suggest a linear dependence between the activity of caspase-1 and the concentration of the VGVAPG peptide after 24 h of exposition. Based on our previously published data, we chose the concentration of 10 nM VGVAPG peptide for further experiments (Szychowski and Gmiński 2019a). Our experiments show that rosiglitazone (a PPARγ agonist) reduces the VGVAPG–peptide-stimulated activity of caspase-1. Moreover, the conducted experiments show that, after 24 h, the VGVAPG peptide decreases both the secretion of IL-1β into the cell-culture medium and the expression of the IL-1βR1 protein in cells. Co-treatment of the astrocytes with the VGVAPG peptide and rosiglitazone potentiated this effect. However, after 48-h astrocyte co-treatment with the VGVAPG peptide and rosiglitazone, we observed an increase in the release of IL-1β into the cell-culture medium, whereas the expression of IL-1βR1 in cells continued to decrease. To date, researchers have described that rosiglitazone can interrupt the positive feedback loop of IL-1β through increased production of IL-1Ra in rat synovial fibroblasts (Moulin et al. 2005). Similarly, in the rat brain with cerebral ischemia, pioglitazone (a PPARγ agonist) reduced the level of IL-1β but upregulated IL-1Ra (Glatz et al. 2010). Moreover, different thiazolidinediones have been found to decrease the induction of pro-inflammatory genes of IL-6 and IL-1β in rats with spinal cord injury (Park et al. 2006).

To date, it is well known that caspase-1 can be activated by increased levels of ROS. Moreover, SOD1 is necessary for the production of mature IL-1β activity (Meissner et al. 2008; Harijith et al. 2014). Due to the crucial role of SOD1 in caspase-1 activation, we decided to measure the expression of SOD1 and CAT in mouse astrocytes. Our experiments show that, after 24- and 48-h exposure to the 10 nM VGVAPG peptide, there was an increase in the expression of SOD1 in astrocytes; however, there was a decrease in the expression of CAT after 24-h exposure. Cell co-stimulation with the VGVAPG peptide and rosiglitazone increased the expression of SOD1 and CAT, compared to the expressions in the group treated with the VGVAPG peptide. Our previously published data show that the VGVAPG peptide increases the ROS production in mouse primary astrocytes and a human neuroblastoma (SH-SY5Y) cell line (Szychowski et al. 2019a; Szychowski and Gmiński 2019a). Furthermore, we observed that, after 24-h exposure of a SH-SY5Y cell line to 50 nM VGVAPG peptide, there was decreased protein expression of SOD1 but the expression of CAT was unaltered (Szychowski et al. 2019a). On the other hand, after 48-h exposure of a SH-SY5Y cell line to 50 nM VGVAPG peptide, there was no change in the expression of SOD1 although there was decreased expression of CAT (Szychowski et al. 2019a). To date, rosiglitazone has been reported to increase the activities of SOD and CAT in the lung tissue of ovoalbumin-sensitized guinea pigs with bronchial asthma (El-Naa et al. 2015). Moreover, rosiglitazone exhibits neuroprotective properties by increasing the activities of CAT, Cu/Zn-SOD, and Mn-SOD in mice with traumatic brain injury (Yi et al. 2008).

On the other hand, several studies have shown that caspase-1 has a regulatory function in cytoprotection, tissue repair, and autophagy in nonimmune cell types, such as keratinocytes, astrocytes, hepatocytes, and cardiomyocytes (Keller et al. 2008; Groß et al. 2012; Sun et al. 2013; Saitoh and Akira 2016). Stimulation of astrocytes by the VGVAPG peptide increases the activity of caspase-1 with a simultaneous decrease in IL-1β secretion, we cannot rule out the function of this caspase in cytoprotection, tissue repair, or autophagy.

Due to the key role of PPARγ and NF-κB in the inflammatory process, the next step of our study was to investigate the expression of this protein in the studied cells. After 24-h exposure of mouse astrocytes to the VGVAPG peptide, the expression of PPARγ was slightly decreased, and this effect was potentiated by rosiglitazone. However, 48-h exposure to the VGVAPG peptide did not affect PPARγ expression compared to the control. In addition, our experiments show that 48-h exposure to the VGVAPG peptide decreased the expression of NF-κB in mouse astrocytes. Moreover, rosiglitazone potentiated the effect of the VGVAPG peptide. Our previously published data showed that PPARγ is involved in the mechanism of action of the VGVAPG peptide in mouse astrocytes and the SH-SY5Y cell line (Szychowski et al. 2019a; Szychowski and Gmiński 2019b). In more recently published research, an unexplained mechanism of action of the VGVAPG peptide causes activation of PPARγ and enhances its expression (Szychowski and Gmiński 2019b). The available data show that the VGVAPG peptide acts in similar/synergistic ways to that of PPARγ agonists in mouse astrocytes and affects the pathways controlled by PPARγ (Szychowski et al. 2019a, b). Studies, to date, have reported that EDPs are involved via NF-κB in the inflammatory response in human melanoma cell lines and *ligamentum flavum* cells (Debret et al. 2006; Chao et al. 2012). Moreover, it is well known that PPARγ agonists and glucocorticoids inhibit the DNA-binding activity of NF-κB and thereby disrupt the NF-κB pathway (Remels et al. 2009; Liu et al. 2017). The PPARγ-induced dysfunction of the NF-κB pathway is the main mechanism of attenuation of the inflammatory process by PPARγ agonists (Kvandová et al. 2016; Scirpo et al. 2016). Therefore, according to our findings that the VGVAPG peptide has properties that stimulate PPARγ activation and expression, we postulate that this may be the mechanism by which inflammatory markers are decreased in mouse astrocytes.

## Conclusion

Our report presents the novel finding that the VGVAPG peptide increases the activity of caspase-1 in astrocytes in vitro. Simultaneously, the VGVAPG peptide decreases IL-1β release into the cell-culture medium from astrocytes*.* An analysis of protein expression using the ELISA method revealed that the VGVAPG peptide increases the protein expression of SOD1 whereas it decreases the expression of IL-1βR1, CAT, and NF-κB. Therefore, the available data suggests that, at a concentration of 10 nM, the VGVAPG peptide acts synergistically with agonists of PPARγ in mouse astrocytes. However, due to the lack of sufficient data to explain the molecular mechanism of action of the VGVAPG peptide in the nervous system, more studies on this topic are necessary.

## Abbreviations

CAT: Catalase
CNS: Central nervous system
DMSO: Dimethyl sulfoxide
EDPs: Elastin-derived peptides
FBS: Fetal bovine serum
ICE: Interleukin-1-beta–converting enzyme
IL-1β: Interleukin-1 beta
IL-1βR1: Interleukin 1 beta receptor 1
MMPs: Matrix metalloproteinases
NF-κB: Nuclear factor kappa-light-chain-enhancer of activated B cells
PBS: Phosphate-buffered saline
PPARγ: Peroxisome proliferator-activated receptor gamma
ROS: Reactive oxygen species
SOD1: Superoxide dismutase 1
T2DM: Type 2 diabetes mellitus
VGVAPG: Gly-Val-Ala-Pro-Gly
